# Characterization of Anti-Phospholipid Antibodies in Lyme Borreliosis Using In-House Developed ELISAs

**DOI:** 10.3390/antib15030051

**Published:** 2026-06-22

**Authors:** Polona Žigon, Katja Lakota, Katarina Ogrinc, Petra Bogovič, Franc Strle

**Affiliations:** 1 Department of Rheumatology, University Medical Centre Ljubljana, 1000 Ljubljana, Slovenia; katja.lakota@kclj.si; 2 The Faculty of Mathematics, Natural Sciences and Information Technologies (UP FAMNIT), University of Primorska, 6000 Koper, Slovenia; 3 Department of Infectious Diseases, University Medical Center Ljubljana, 1000 Ljubljana, Slovenia; katarina.ogrinc@kclj.si (K.O.); petra.bogovic@kclj.si (P.B.); franc.strle@kclj.si (F.S.)

**Keywords:** Lyme borreliosis, antiphospholipid antibodies, anti-phosphatidylserine antibodies, anti-phosphatidic acid antibodies, anti-phosphatidylcholine antibodies, *Borrelia burgdorferi* sensu lato

## Abstract

Objectives: *Borrelia burgdorferi* sensu lato, a spirochete bacterium responsible for Lyme borreliosis—the most common tick-borne infection in North America and Europe—can trigger the production of antiphospholipid antibodies. These antibodies target host lipids such as cardiolipin (CL), phosphatidic acid (PA), phosphatidylcholine (PC), and phosphatidylserine (PS), which the spirochete incorporates into its membrane from the surrounding environment. Although antiphospholipid antibodies are typically associated with antiphospholipid syndrome (APS), they may also arise during infections, including Lyme borreliosis. This study aimed to develop and optimize several enzyme-linked immunosorbent assays (ELISAs) for measuring various antiphospholipid antibodies in patients with Lyme borreliosis. Methods: Thirty patients diagnosed with Lyme borreliosis were enrolled: ten with solitary erythema migrans (EM), ten with multiple EM (MEM), and ten with late manifestations known as acrodermatitis chronica atrophicans (ACA). Forty healthy blood donors served as controls. Four distinct antiphospholipid antibody ELISAs were developed, each using a different phospholipid coating: CL, PA, PC, and PS. Serum of APS patient was used as a positive control and for standard curve generation. Results: All four ELISAs were successfully established and demonstrated good measurement precision. Significant differences in antiphospholipid antibody levels and positivity rates were observed between Lyme borreliosis patients and healthy blood donors. Notably, levels of antibodies directed against PA (aPA), PC (aPC), and PS (aPS), both IgG and IgM, were significantly higher in patients with late Lyme borreliosis, manifested as ACA, compared to healthy blood donors. In contrast, anti-CL (aCL) levels did not differ significantly between groups. Patients with ACA also showed the highest frequency of multiple antiphospholipid antibody positivity, with 7 out of 10 patients testing positive for three or more antiphospholipid antibodies. Conclusions: Accurate and precise in-house ELISAs for the detection of aCL, aPA, aPC, and aPS using APS sera as standard material were developed and validated for the analysis of samples of patients with Lyme borreliosis. Our data suggest that antiphospholipid antibody levels—specifically aPA, aPC, and aPS—differ across clinical manifestations of Lyme borreliosis, with the greatest increases observed in patients with ACA.

## 1. Introduction

Antiphospholipid antibodies are a heterogeneous group of autoantibodies directed against phospholipids or phospholipid-binding proteins, most notably β2 glycoprotein I and cardiolipin. Their persistent presence in serum is associated with an increased risk of thrombosis in various autoimmune diseases and forms the basis of the laboratory criteria for antiphospholipid syndrome (APS) [1,2]. Although antiphospholipid antibodies are now strongly linked to APS, they were originally described in the context of infectious diseases. The first observations came from patients infected with *Treponema pallidum*, where cardiolipin-based serological tests for syphilis revealed reactivity that was later recognized as antiphospholipid antibodies [3]. Continued use of these assays eventually showed that some patients with autoimmune diseases, particularly systemic lupus erythematosus, exhibited “false positive” results, which led to the discovery of pathogenic antiphospholipid antibodies and the later conceptualization of anticardiolipin syndrome and APS [4,5].

Beyond syphilis, transient antiphospholipid antibody production has been documented in a range of bacterial, viral, and parasitic infections. In these contexts, antiphospholipid antibodies are thought to arise due to infection-induced immune activation, molecular mimicry, and the exposure of phospholipid antigens during inflammation [6,7].

Similar to *Treponema pallidum*, infection with the causative agents of Lyme borreliosis (*Borrelia burgdorferi* sensu lato, also referred to as Lyme borreliae) has also been shown to induce the production of antiphospholipid antibodies that recognize host phospholipids. This phenomenon was first systematically demonstrated by Gwynne and co-authors, who proposed their potential utility as infection-associated biomarkers in Lyme borreliosis [8]. Lyme borreliosis is the most common tick-borne disease in the Northern hemisphere and manifests with a wide and evolving range of clinical features [9]. In the absence of antibiotic therapy, disease progression typically follows three stages, beginning with early localized skin infection presenting as erythema migrans (EM), followed by early disseminated disease such as multiple erythema migrans (MEM) or Lyme neuroborreliosis and culminating in late manifestations, of which acrodermatitis chronica atrophicans (ACA) is the most characteristic in Europe.

Diagnosing Lyme borreliosis remains challenging [10]. Clinical symptoms may be non-specific or atypical, especially in disseminated and late disease, where systemic manifestations can mimic other inflammatory or autoimmune conditions. Laboratory diagnosis relies primarily on serological tests detecting anti *Borrelia* antibodies. However, serology is insensitive during the first weeks of infection, when most patients present with EM. Antibody responses may be delayed, leading to false negative results early in the disease course, and antibodies can persist long after bacterial clearance, limiting their usefulness for assessing disease activity or treatment response. Thus, current serological testing cannot reliably distinguish active from past infection or evaluate antibiotic treatment efficacy, creating a diagnostic gap both in early disease and during follow up.

From a pathophysiological perspective, *Borrelia* lack complete biosynthetic pathways for several membrane lipids and therefore scavenge host-derived phospholipids and cholesterol, incorporating them into their outer membrane. Because Lyme borrelia is unable to synthesize its own fatty acids, its lipid composition mirrors that of host tissues. Lipid exchange between the bacterium and eukaryotic cell membranes occurs through direct contact or via outer membrane vesicles [11]. Several of these host-derived phospholipids—such as phosphatidylserine (PS), phosphatidylcholine (PC), and phosphatidic acid (PA)—may be recognized by antiphospholipid antibodies that emerge early in infection. Previous studies in a small American cohort demonstrated that antibody responses to host phospholipids, measured using an antiphospholipid antibodies ELISA, may outperform standard diagnostic tests in the context of Lyme borreliosis [8]. The primary objective of this study was therefore to develop an in-house ELISA for the detection of various antiphospholipid antibodies and to evaluate their diagnostic performance in a European cohort of patients with early and late Lyme borreliosis.

## 2. Materials and Methods

### 2.1. Participants

Serum samples from 30 patients with Lyme borreliosis, of which 10 were presented with early localized stage—EM, 10 with early disseminated stage—MEM, and 10 with late disseminated stage—ACA, were collected at the Department of Infectious Diseases, University Medical Centre Ljubljana, Slovenia (Table 1). Additionally, blood samples from 40 healthy blood donors (HBD) were collected. The study was performed according to the guidelines of the Declaration of Helsinki and approved by the National Medical Ethics Committee, Ljubljana, Slovenia (0120-571/2024-2711-3, date of approval 15 January 2025).

### 2.2. Serum Collection

Samples were collected in routine clinical settings. Blood samples were centrifuged at 1800× *g* for 10 min within 2 h of collection, and serum was separated directly from the cell pellet. Samples were stored at 4 °C and analyzed the next day or aliquoted into tubes and stored at −80 °C for later analysis.

### 2.3. In-House ELISAs for the Measurement of Antiphospholipid Antibodies

**Figure 1:**
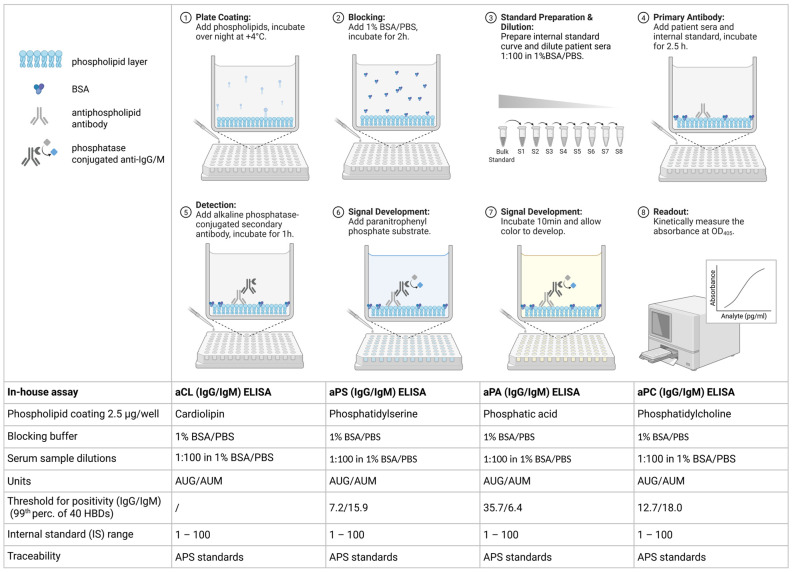
Characteristics of aCL, aPA, aPC, and aPS ELISA. aCL, anti-cardiolipin antibodies; aPA, anti-phosphatidic acid antibodies; aPC, anti-phosphatidylcholine antibodies; aPS, anti-phosphatidylserine antibodies; BSA, bovine serum albumin; APS, antiphospholipid syndrome. Created in BioRender. Žigon, P. (2026) https://BioRender.com/4zqste7 (accessed on 5 May 2026).

Four anti-phospholipid antibodies, including anti-cardiolipin (aCL), anti-phosphatidic acid (aPA), anti-phosphatidylcholine (aPC), and anti-phosphatidylserine (aPS) of IgG and IgM isotypes, were measured using in-house ELISAs (Figure 1). These assays followed the protocol first described in 1997 [12], subsequently evaluated in several studies [13,14], and adapted according to the method published by Gwynne et al. [8]. Polystyrene microliter plates (Costar medium binding EIA/RIA plates, Cambridge, MA, USA) were coated with 2.5 µg/well of cardiolipin, phosphatidylserine, phosphatidylcholine, or phosphatidic acid (Avanti Polar Lipids, Inc., Alabaster, AL, USA) in absolute ethanol and dried overnight at 4 °C. All subsequent steps were performed at room temperature. Wells were blocked for 2 h with 1% bovine serum albumin (BSA) in phosphate-buffered saline (PBS). After washing three times with PBS, patient sera diluted 1:100 in 1% BSA/PBS were applied in duplicate and incubated for 2.5 h. Plates were then washed four times with PBS containing 0.05% Tween 20, followed by the addition of alkaline phosphatase-conjugated goat anti-human IgG or IgM antibodies (Accurate Chemical & Scientific Corporation, Carle Place, NY, USA) and a 60 min incubation. After another four washes, 100 mL/well of para-nitrophenyl phosphate (Sigma Chemical Company, St. Louis, MO, USA) dissolved at 1 g/L in 1 M diethanolamine buffer (pH 9.8), was applied. The OD_405_ was measured kinetically using a spectrometer until optimal alignment with the predicted values of the internal standards (IS) was achieved.

### 2.4. Verification of the ELISA Tests

Precision (repeatability and intermediate precision) of the ELISAs was evaluated in accordance with the Clinical and Laboratory Standards Institute (CLSI) EP5-A3 recommendations. A positive control sample was tested in duplicate across four separate runs to assess within-run and between-run variability. To find significant outliers among the results, the Grubbs’ test was performed.

### 2.5. Statistical Analysis

Group comparisons were performed using one-way ANOVA followed by Tukey’s multiple comparison test. Correlation analyses were conducted using Pearson’s correlation test. The diagnostic value of different antiphospholipid antibodies was determined by ROC curve analysis. Data processing and statistical analyses were performed using GraphPad Prism 10.3.1 software (GraphPad Software, San Diego, CA, USA). A *p*-value of less than 0.05 was considered statistically significant.

## 3. Results

### 3.1. Verification of the aCL, aPA, aPC and aPS ELISA Tests

To evaluate the measurement precision of aCL IgG/IgM, aPA IgG/IgM, aPC IgG/IgM, and aPS IgG/IgM ELISAs, within-run and between-run imprecision was assessed by testing a positive sample in duplicate across four independent runs (Supplementary Table S1). Within-run coefficients of variation (%CV) ranged from 2.39% to 8.80% for IgG, and from 4.45% to 7.81% for IgM. Between-run %CV ranged from 7.20% to 17.30% for IgG, and from 4.09% to 17.30% for IgM. All eight ELISAs showed low intra-assay variability (2.39–8.80%) and acceptable inter-assay variability (4.09–17.30%), confirming robust analytical performance.

### 3.2. Levels of Antiphospholipid Antibodies

**Figure 2:**
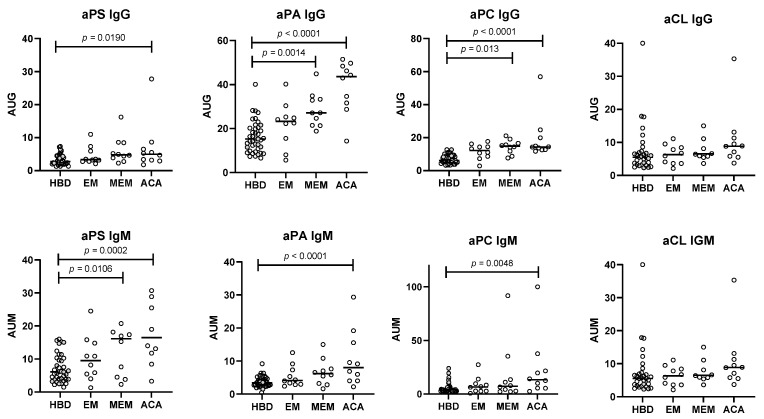
Levels of antiphospholipid antibodies in patients with Lyme borreliosis and healthy blood donors. Group comparisons were performed using one-way ANOVA followed by Tukey’s multiple comparison test. The horizontal lines represent median values. aCL: anti-cardiolipin antibodies; aPA: anti-phosphatidic acid antibodies; aPC: anti-phosphatidylcholine antibodies, aPS: anti-phosphatidylserine antibodies; AUG: arbitrary units IgG; AUM: arbitrary units IgM; EM: erythema migrans; MEM; multiple erythema migrans; ACA: acrodermatitis chronica atrophicans; HBD: healthy controls.

Both IgG and IgM levels of aPA, aPC, and aPS were significantly higher in patients with late disseminated Lyme borreliosis—ACA compared to HBDs. In patients with MEM IgG aPA and aPC, as well as IgM aPS levels, were also significantly elevated compared to HBDs. In contrast, anti-cardiolipin (aCL) antibody levels did not differ significantly between the groups (Figure 2).

### 3.3. Correlation Between Different Antiphospholipid Antibodies

**Figure 3:**
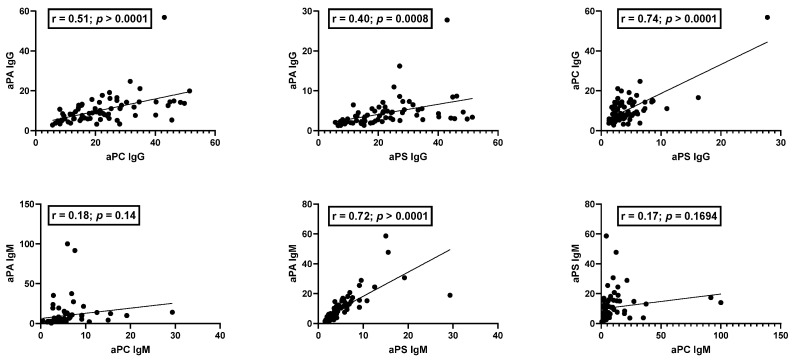
Correlation coefficients between aPS, aPA and aPC antibodies, shown separately for IgG and IgM isotypes. r: Pearson correlation coefficient, aPS: anti-phosphatidylserine antibodies; aPA: anti-phosphatidic acid antibodies; aPC: anti-phosphatidylcholine antibodies.

A positive linear correlation was observed among the IgG isotypes of aPA, aPC, and aPS antibodies. Specifically, the correlation coefficients were r = 0.51 for aPA vs. aPC (*p* < 0.0001), r = 0.40 for aPA vs. aPS (*p* = 0.0008), and r = 0.74 for aPC vs. aPS (*p* < 0.0001) (Figure 3). For the IgM isotypes, aPA IgM showed a significant positive correlation with aPS IgM, whereas no significant correlations were detected between aPA IgM and aPC IgM or between aPC IgM and aPS IgM (Figure 3).

### 3.4. Positivity of Antiphospholipid Antibodies in Lyme Borreliosis Patients

The threshold for individual antiphospholipid antibody positivity was determined using a non-parametric approach, based on the 99th percentile of values measured in a group of 40 HBDs, as the distribution of antiphospholipid antibody levels was not normal. Individual values, calculated thresholds, and the number of positive HBDs are presented in Table 2. Significant outliers were identified for aPA IgG/IgM and aPC IgM using Grubbs’ test, whereas no outliers were detected for aPS IgG/IgM or aPC IgG. Importantly, none of the healthy blood donors tested positive for more than one antiphospholipid antibody.

Patients with Lyme borreliosis exhibited a significantly higher overall frequency of antiphospholipid antibody positivity compared with healthy blood donors (Table 3). Among the clinical subgroups, the highest prevalence was observed in patients with ACA, where 7 out of 10 individuals tested positive for three or more antiphospholipid antibodies. In the MEM group, 6 out of 10 patients showed positivity for three or more antibodies, whereas in the EM group, this applied to 3 patients.

### 3.5. ROC Curve Analysis and Diagnostic Applicability of the Tested Antiphospholipid Antibodies

**Figure 4:**
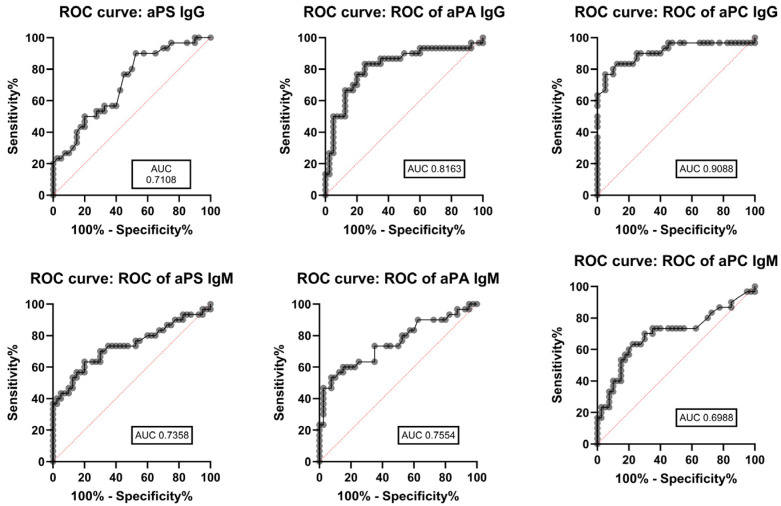
ROC analysis: comparison of area under the curve (AUC) between aPS G/M. aPA G/M and aPC G/M ELISAs. Red dashed line indicates the line of no discrimination (AUC = 0.5).

The diagnostic performance of the individual ELISAs for Lyme borreliosis was evaluated using ROC curve analysis (Figure 4). The areas under the curve (AUC) for the different assays ranged from 0.6988 to 0.9088. Among all tested antiphospholipid antibodies, aPC IgG demonstrated the highest diagnostic applicability for distinguishing patients with Lyme borreliosis from healthy controls, based on its AUC value.

## 4. Discussion

In the present study, we have expanded the currently limited knowledge on antiphospholipid antibodies in Lyme borreliosis, which has so far largely relied on a single primary study by Gwynne et al. and its subsequent commentary [8,15]. Consistent with their findings, we confirmed that infection with *Borrelia burgdorferi* is associated with the induction of antibodies against phosphatidic acid, phosphatidylcholine, and phosphatidylserine, whereas anti-cardiolipin antibodies do not appear to be significantly elevated. This supports the concept that lipid scavenging by *Borrelia* contributes to the generation of a distinct antiphospholipid antibody profile. The absence of anti-cardiolipin antibodies represents an important distinction between Lyme borreliosis and syphilis. In syphilis, infection with *Treponema pallidum* has been shown to induce the release of mitochondrial cardiolipin from host cells, thereby promoting the generation of aCL antibodies [16]. In contrast, Lyme borreliae does not appear to elicit a comparable cardiolipin-directed immune response, indicating differences in host–pathogen interactions and lipid-antigen exposure.

Importantly, our study extends the observations from Gwynne et al. in several key aspects. First, while previous work primarily focused on IgG responses, we systematically analyzed both IgG and IgM isotypes. We demonstrated that IgM antiphospholipid antibodies are also significantly elevated, particularly in patients with more advanced disease (ACA). Although persistent IgM responses are not typically expected in late-stage disease, several mechanisms described in *Borrelia* infection may account for this finding. Chronic manifestations such as ACA are characterized by persistent local inflammation and ongoing antigenic stimulation, which can maintain IgM production. In addition, *Borrelia burgdorferi* is known to induce polyclonal B-cell activation and to expose host-derived phospholipids through its lipid-scavenging biology, both of which may contribute to sustained IgM reactivity. Taken together, the inclusion of IgM provides additional insight into the temporal dynamics of the antiphospholipid antibody response in Lyme borreliosis, which was not addressed in the initial study.

Second, unlike the study by Gwynne et al., which included limited or heterogeneous patient cohorts, we analyzed well-defined clinical subgroups of Lyme borreliosis, encompassing early localized (EM), early disseminated (MEM), and late disease (ACA). This allowed us to demonstrate a clear gradient in antiphospholipid antibody levels and positivity rates across disease stages, with the highest levels observed in ACA. Moreover, the presence of multiple antiphospholipid antibodies was markedly more frequent in late disease, supporting the hypothesis that cumulative exposure to *Borrelia*-derived or host-derived phospholipids enhances the breadth of the antibody response.

Third, an important difference between our study and previous work lies in the studied population. While Gwynne et al. investigated a cohort from the United States, our study was conducted in a European population. This is particularly relevant, as differences in genetic background, environmental exposures, and circulating *Borrelia* species across geographical regions may influence the immune response and the profile of antiphospholipid antibodies. Therefore, our findings provide important validation and extension of previous observations in a distinct population setting.

Fourth, our study includes a larger and better-characterized cohort compared to the initial report, addressing one of the key limitations highlighted in subsequent commentary, where validation in larger patient populations was explicitly recommended. By increasing the sample size and including a control group of healthy blood donors, we were able to define robust cut-off values using a non-parametric approach and reduce the risk of false-positive interpretations. A further strength of our study lies in the standardization of the in-house ELISAs. We employed a serum sample from a patient with antiphospholipid syndrome as a reference standard, allowing antibody levels to be expressed in arbitrary units. This approach enabled direct comparison across different ELISA runs and reduced inter-assay variability, thereby improving the reliability and reproducibility of the measurements. Such standardization represents an important methodological advantage over a previous study, where variability between assays may have limited the comparability of subsequent results.

A major strength of our study is the evaluation of the diagnostic performance of individual antiphospholipid antibodies using ROC curve analysis. While previous studies suggested a potential biomarker role for these antibodies, they did not formally assess their diagnostic accuracy. In our cohort, several antibodies demonstrated good discriminatory capacity, with aPC IgG showing the highest AUC. These findings indicate that antiphospholipid antibodies—particularly when assessed as a panel—may have clinically relevant diagnostic value in distinguishing Lyme borreliosis patients from healthy individuals.

In addition, we performed a detailed correlation analysis between different antiphospholipid antibodies. We observed strong correlations among IgG isotypes (especially between aPC and aPS), suggesting a coordinated immune response targeting structurally or functionally related phospholipids. In contrast, IgM responses showed weaker and more selective correlations, which may reflect differences in antigen exposure, affinity maturation, or B-cell activation pathways. These findings provide novel insight into the immunological relationships between different antiphospholipid specificities.

Several limitations should be acknowledged. The sample size, although larger than in the study by Gwynne et al. [8], remains modest, and longitudinal data were not available to assess the persistence of antiphospholipid antibodies following treatment. Previous studies have suggested that these antibodies may decline after antibiotic therapy, indicating their potential role as markers of disease activity. Future studies should therefore include longitudinal sampling and comparisons with other infectious and autoimmune conditions to better define specificity and clinical relevance.

In conclusion, our study validates and significantly extends previous findings on antiphospholipid antibodies in Lyme borreliosis. By including both IgG and IgM isotypes, analyzing distinct clinical subgroups, increasing cohort size, and applying ROC and correlation analyses, we provide a more comprehensive characterization of these antibodies.

## 5. Conclusions

We developed and standardized in-house ELISAs for aCL, aPA, aPC, and aPS using APS reference serum, enabling inter-assay comparability. Patients with acrodermatitis chronica atrophicans showed significantly elevated levels of IgG and IgM aPS, aPA, and aPC compared to healthy controls. Testing multiple antiphospholipid antibodies proved to be the most effective approach for distinguishing between subgroups of patients with Lyme borreliosis. ROC analysis demonstrated meaningful diagnostic potential, with IgG aPC showing the highest discriminatory performance. Overall, our findings support the use of aPC, aPA, and aPS as adjunct biomarkers in Lyme borreliosis, particularly when assessed as a panel and interpreted in the clinical context.

## Figures and Tables

**Table 1:** Baseline demographic, serological, and clinical characteristics of study participants.

		Healthy Blood Donors (HBD, n = 40)	Solitary Erythema Migrans (EM, n = 10)	Multiple Erythema Migrans (MEM, n = 10)	Acrodermatitis Chronica Atrophicans (ACA, n = 10)
Age	Median (IQR)	45 (12)	56 (26)	50 (20)	75 (8)
Gender	M/F (%)	78/22	60/40	40/60	10/90
Skin culture for BB	n (% positive)	/	1/7 (14.3)	2/9 (22.2)	0/10 (0)
*Borrelia* antibodies IgG	n (% positive)	/	7 (70)	9 (90)	10 (100)
*Borrelia* antibodies IgM	n (% positive)	/	1 (10)	9 (90)	7 (70)

**Table 2:** Levels of aPS, aPA, and aPC in healthy blood donors.

	aPS		aPA		aPC		No. of Positive aPL
	IgG [AUG]	IgM [AUG]	IgG [AUG]	IgM [AUG]	IgG [AUG]	IgM [AUG]	
HBD 1	3.4	7.1	24.6	0.5	6.1	3.4	0
HBD 2	4.5	3.9	28.1	4.1	3.3	3.7	0
HBD 3	7.1	15.2	21.6	5.4	10.1	13.2	0
HBD 4	5.5	3.8	17.9	2.9	8.7	4.4	0
HBD 5	2.1	4.1	14.8	3.1	5.1	3.7	0
HBD 6	2.0	8.0	10.8	4.1	4.4	3.5	0
HBD 7	3.2	15.6	15.3	9.17 **	12.7	10.4	1
HBD 8	1.7	6.1	8.8	3.0	5.1	5.7	0
HBD 9	3.9	4.5	16.4	3.6	6.4	6.3	0
HBD 10	2.3	9.6	7.4	4.3	4.3	3.6	0
HBD 11	6.1	7.6	16.9	3.5	8.4	2.3	0
HBD 12	1.9	9.4	11.5	6.36 *	8.3	10.6	1
HBD 13	3.6	3.7	12.4	2.7	8.2	23.9 **	1
HBD 14	2.8	4.6	19.9	1.9	11.1	2.9	0
HBD 15	4.5	2.6	13.3	2.8	9.5	2.8	0
HBD 16	1.9	5.7	17.4	2.4	5.9	2.6	0
HBD 17	2.9	6.4	9.7	4.1	5.9	7.6	0
HBD 18	2.0	14.7	14.2	3.7	10.7	5.5	0
HBD 19	1.3	3.4	15.3	2.5	5.9	2.9	0
HBD 20	4.8	5.4	21.7	2.7	5.6	2.3	0
HBD 21	3.7	2.6	20.4	3.4	3.2	4.2	0
HBD 22	2.4	3.0	15.5	2.7	7.6	2.2	0
HBD 23	5.9	6.7	20.2	2.7	9.4	19.5 *	1
HBD 24	4.9	4.0	23.0	2.6	8.0	2.7	0
HBD 25	2.4	5.4	19.0	3.7	8.9	3.1	0
HBD 26	2.5	12.4	27.3	4.5	5.2	2.3	0
HBD 27	1.3	3.2	6.6	2.3	3.7	2.8	0
HBD 28	2.4	4.9	8.7	2.6	4.9	2.9	0
HBD 29	2.3	11.4	14.4	6.36 *	12.7	6.7	1
HBD 30	6.5	7.4	11.7	4.1	3.8	6.1	0
HBD 31	3.7	16.0 *	12.9	5.6	6.0	2.5	1
HBD 32	1.3	10.3	7.4	3.5	4.9	3.8	0
HBD 33	2.0	10.3	18.2	5.0	6.1	2.5	0
HBD 34	4.3	11.5	40.1 *	5.5	7.8	4.4	1
HBD 35	7.3 *	15.0	28.2	5.2	11.1	15.4	1
HBD 36	2.2	2.2	9.4	2.1	7.4	2.5	0
HBD 37	2.5	6.2	12.7	2.3	5.9	2.3	0
HBD 38	4.5	2.5	24.4	2.1	8.3	2.8	0
HBD 39	3.0	6.1	45.5 **	3.7	5.4	2.6	1
HBD 40	2.0	1.5	8.2	1.5	3.4	1.8	0
Mean	3.3	6.9	16.6	3.5	7.0	4.9	
99th perc.	6.87	15.48	35.71	6.36	12.73	17.95	

* Positive samples; ** outliers, aPA: anti-phosphatidic acid antibodies; aPC: anti-phosphatidylcholine antibodies; aPS: anti-phosphatidylserine antibodies; HBD: healthy blood donors.

**Table 3:** Levels of aPA, aPC, and aPS in Lyme borreliosis patients.

	aPS		aPA		aPC		No. of Positive aPL
	IgG [AUG]	IgM [AUG]	IgG [AUG]	IgM [AUG]	IgG [AUG]	IgM [AUG]	
EM 1	3.2	5.8	24.1	2.8	7.3	2.7	0
EM 2	7.3 *	10.9	30.4	9.1 *	14.3 *	9.6	3
EM 3	3.2	14.8	22.5	7.3 *	8.6 *	27.4 *	3
EM 4	3.4	5.4	40.2 *	3.4	14.5 *	2.6	2
EM 5	2.8	24.5 *	24.8	12.6 *	16.2 *	13.9	3
EM 6	2.8	8.2	8.1	4.3	10.7	2.8	0
EM 7	2.1	1.3	5.7	2.4	2.8	0.6	0
EM 8	5.9	10.8	22.3	4.0	17.7 *	8.3	1
EM 9	11.0 *	15.8	25.3	5.3	11.0	6.3	1
EM 10	3.4	4.0	15.5	3.0	13.2 *	6.9	1
mean	3.4	8.6	19.8	3.6	8.0	6.0	
No. of positives	2	1	1	3	5	1	
MEM 1	4.4	2.3	21.5	1.6	8.7	2.8	0
MEM 2	8.4 *	4.5	44.9 *	3.2	14.5 *	6.6	3
MEM 3	5.2	17.4 *	33.3	7.6 *	7.6	91.7 *	3
MEM 4	3.8	15.3	32.9	10.8 *	11.8	2.3	1
MEM 5	2.3	17.0 *	21.3	5.5	14.2 *	2.3	2
MEM 6	8.6 *	58.6 *	27.2	15.0 *	15.2 *	4.3	4
MEM 7	4.6	20.8 *	24.7	7.0 *	19.1 *	11.0	3
MEM 8	4.9	18.2 *	18.8	6.5 *	15.6 *	7.9	3
MEM 9	2.8	7.6	34.9	5.8	21.1 *	13.4	1
MEM 10	16.2 *	3.8	27.1	2.8	16.6 *	35.3 *	3
mean	4.0	6.7	26.8	3.8	9.4	6.3	
No. of positives	3	5	1	5	7	2	
ACA 1	8.7 *	30.7 *	46.2 *	19.2 *	14.9 *	10.1	5
ACA 2	4.7	3.3	48.4 *	2.2	14.2 *	2.4	2
ACA 3	5.5	19.0 *	34.6	29.4 *	14.4 *	14.1	3
ACA 4	3.4	47.7 *	51.5 *	15.6 *	19.9 *	12.5	4
ACA 5	27.8 *	14.0	43.0 *	6.0	56.9 *	100.0 *	4
ACA 6	3.2	25.5 *	44.3 *	9.1 *	12.6	5.5	3
ACA 7	2.9	28.9 *	49.7 *	9.5 *	13.7 *	21.6 *	5
ACA 8	6.5	13.1	31.6	6.9 *	24.7 *	37.5 *	3
ACA 9	1.8	11.8	14.3	4.0	11.7	5.3	0
ACA 10	5.3	8.5	28.7	4.0	12.7	19.6	0
mean	4.1	10.1	27.3	4.1	12.3	9.9	
No. of positives	2	5	6	6	7	3	

* Positive samples; aPA: anti-phosphatidic acid antibodies; aPC: anti-phosphatidylcholine antibodies; aPS: anti-phosphatidylserine antibodies; EM: erythema migrans; MEM: multiple erythema migrans; ACA: acrodermatitis chronica atrophicans.

## Data Availability

The original contributions presented in this study are included in the article/Supplementary Material. Further inquiries can be directed to the corresponding author.

## Supplementary Materials

The following supporting information can be downloaded at https://www.mdpi.com/article/10.3390/antib15030051/s1. Table S1: The measurement precision of aCL, aPA, aPC, and aPS (IgG and IgM) ELISAs.

## Abbreviations

The following abbreviations are used in this manuscript:

aPA	Anti-phosphatidic acid antibodies
aPC	Anti-phosphatidylcholine antibodies
aPS	Anti-phosphatidylserine antibodies
HBD	Healthy blood donors
AUG	Arbitrary units IgG
AUM	Arbitrary units IgM
EM	Erythema migrans
MEM	Multiple erythema migrans
ACA	Acrodermatitis chronica atrophicans
