# Supplementary material for: Characterization of Anti-Phospholipid Antibodies in Lyme Borreliosis Using In-House Developed ELISAs
Source: Antibodies (Basel). 2026 Jun 22;15(3):51. doi: 10.3390/antib15030051 (PMC13296186; doi:10.3390/antib15030051)
Supplement: Supplementary file 1 [file antibodies-15-00051-s001.zip › antibodies-4331194-supplementary.pdf]

**Supplemental Table S1:** The measurement precision of aCL, aPA, aPC, and aPS (IgG and IgM) ELISAs

| | aCL IgG ELISA | aCL IgM ELISA | aPA IgG ELISA | aPA IgM ELISA | aPC IgG ELISA | aPC IgM ELISA | aPS IgG ELISA | aPS IgM ELISA |
|---|---|---|---|---|---|---|---|---|
| *Within-run CV (%)* | 8.80 | 5.40 | 5.00 | 7.81 | 2.39 | 4.45 | 4.39 | 5.92 |
| *Between-run CV (%)* | 17.30 | 17.20 | 11.35 | 12.31 | 7.20 | 6.55 | 15.35 | 4.09 |
